# Health and Well-Being Among College Students in the United States During the COVID-19 Pandemic: Daily Diary Study

**DOI:** 10.2196/45689

**Published:** 2024-08-23

**Authors:** Stephanie T Lanza, Courtney Whetzel, Sandesh Bhandari

**Affiliations:** 1 Department of Biobehavioral Health The Pennsylvania State University University Park, PA United States; 2 The Edna Bennett Pierce Prevention Research Center The Pennsylvania State University University Park, PA United States

**Keywords:** daily diary, college student, young adult, mental health, substance use, stress, well-being, belonging

## Abstract

**Background:**

There is evidence that anxiety and stress increased among college students during the COVID-19 pandemic. However, less is known about daily experiences of affect, worry, substance use behaviors, experiences of pleasure, concern over food security, experiences of bias or discrimination, feelings of belongingness, and other indicators of well-being and how they vary across days in this population.

**Objective:**

This study surveyed a wide range of indicators of health and well-being in daily life over 21 days with a sample of college students in a large university system in the United States during the pandemic. The overall variance in each daily measure was partitioned to reflect the proportion due to (1) between-person differences versus (2) within-person, day-to-day variability. This is important because measures that vary primarily due to between-person differences may be more amenable to interventions that target particular students, whereas measures that vary more due to day-to-day variability may be more amenable to interventions that target day-level contextual factors.

**Methods:**

A sample of 2068 young adult college students (aged 18-24, mean 19.8, SD 1.3 years; 66.6% women) completed a baseline survey; 97.3% (n=2012) then completed up to 21 consecutive daily surveys that assessed a comprehensive set of daily markers of health, behavior, and well-being. The daily diary study produced a total of 33,722 person-days.

**Results:**

Among all person-days, a minority were substance use days (eg, 14.5% of days involved alcohol use, 5.6% vaping, and 5.5% cannabis). Experiences of pleasure were reported on most (73.5%) days. Between-person differences explained more than 50% of the variance in numerous indicators of health and well-being, including daily vaping, cannabis use, other illicit substance use, experiences of bias or discrimination, positive affect, negative affect, worry, food insecurity, and feelings of belonging at the university. In contrast, within-person differences explained more than 50% of the variance in daily alcohol use, cigarette use, stress, experiences of pleasure, where the student slept last night, and physical activity.

**Conclusions:**

College student health and well-being are multifaceted, with some aspects likely driven by person-level characteristics and experiences and other aspects by more dynamic, contextual risk factors that occur in daily life. These findings implicate services and interventions that should target individual students versus those that should target days on which students are at high risk for poor experiences or behaviors.

## Introduction

College students’ well-being is multifaceted, including mental health, substance use behavior, stress, belonging, flourishing, and more. In terms of mental health, anxiety has been the most common problem reported by students [1]. Further, the prevalence of many substance use risk behaviors peaks during college ages, including 35% of young adults reporting past 30-day binge drinking (4+ and 5+ drinks in a row for females and males, respectively) [2]. This population also experiences high rates of acute consequences from alcohol use, including sexual assault and blacking out [3].

Much recent evidence shows that mental health problems among college students increased substantially during the COVID-19 pandemic [4,5]. However, while social isolation, anxiety, and depression increased during the pandemic, certain factors, such as a sense of belonging with their college, seem to be protective against the effects of the pandemic on key health outcomes, particularly among underrepresented racial or ethnic minority and first-generation students [6]. When considering college student health and well-being more broadly, a longitudinal study during the pandemic identified a large subgroup of students with either very low mental and behavioral health risk or elevated mental health symptoms but no other risks (eg, low rates of substance use, sexual activity, physical inactivity, and food insecurity) and a set of 4 smaller subgroups of students with particular configurations of problematic health and well-being, such as the intersection of elevated depression symptoms, recent cannabis use, and recent sexual activity [7].

A more intensive surveillance study of college students can shed important light on the dynamic nature of health and well-being by elucidating both individual characteristics and dynamic, contextual factors that may be linked to multiple facets of student physical and mental health and well-being. Toward this goal, the aim of the present study was to conduct a large daily diary study of college student experiences at the main campus of a large university in the Northeastern United States throughout the 2021 calendar year when the pandemic was still of great concern but students had fully returned to campus for primarily in-person learning. We sought to determine whether a broad range of daily indicators of health and well-being were attributable more to between-person differences or to day-to-day variability, which might indicate greater sensitivity to dynamic and contextualized experiences.

## Methods

### Recruitment and General Procedures

Data for this observational study were collected via web-based surveys from students at a large public university in spring 2021 and fall 2021 as part of the Student Engagement, Learning, and Flourishing study (ie, Project SELF). Over 35,000 undergraduate participants were invited to take part in the study. The inclusion criteria were that participants were full-time undergraduate students at this campus and aged 18-24 years.

Participants were sent the first recruitment email on a Tuesday, with a follow-up (if they had not yet completed the baseline survey) 1 week later. Undergraduate emails were separated into batches and uploaded into REDCap (Research Electronic Data Capture; REDCap Consortium). Participants received individualized survey links in recruitment emails and verified their school email accounts before proceeding. Once a participant with their unique student ID had completed the survey, they were no longer able to fill out another survey. Recruitment emails were staggered, with approximately 2700 students contacted per week, so that experiences throughout the spring and fall semesters would be captured. A total of 9 rounds of emails were sent in the spring 2021 semester, and 9 additional rounds were sent in the fall 2021 semester. Additionally, any spring 2021 nonresponders were given one additional opportunity to participate that fall. All questions were mandatory to answer before moving on to the next question, but every question had a “prefer not to answer” option. The survey was determined to be “complete” when the participant clicked the “submit” button at the end. In total, 2 automatic reminders were sent from REDCap if participants had provided consent and started the questionnaire but had not clicked the “submit” button. Once participants moved on to the next page of the survey, they were not able to go back and change an answer.

Surveys were comprised of multiple scales gathered from collaborating faculty across the university. After compilation and programming, the surveys were extensively tested for branching completeness and automation fluidity. Ongoing spot testing throughout the rollout process ensured that the recruitment and survey responses were imported correctly in REDCap.

Once participants completed the baseline survey, they entered their phone number to receive SMS text messages for the daily diary portion of the study. Daily diary prompts began on the day following baseline survey completion, sent as both an SMS text message and an email at 9 AM EST, with a reminder message 1 hour later to participants who had not yet completed that day’s survey. To be considered valid, participants had to initiate the daily survey before midnight and complete the survey before 1:00 AM EST. The web-based consent and baseline survey were delivered securely via REDCap, which also coordinated all SMS text message and email survey invitations. For the daily diary portion of the study, REDCap invitations pushed out Qualtrics daily survey links.

A baseline survey was completed by 2068 young adult college students; 97.3% (n=2012) then participated in a 21-day diary study. All recruitment, consent, text messaging, reminders for the daily diary, and incentive payments were completed remotely.

### Ethical Considerations

This research was reviewed and approved by the Pennsylvania State University’s institutional review board (approval number: STUDY00015710). Prior to completing the baseline survey, participants completed the informed consent process digitally. This contained all the consent elements: purpose of study, typical length of time to complete surveys, data storage and security, potential risks to participants, and contact information for the principal investigator and institutional review board. Participants had an opportunity to download and print the consent form. Data from the baseline and daily surveys were linked using numeric identifiers; personal identifying information was removed prior to data analysis.

Participants were compensated US $15 for completing the baseline survey and US $2 per daily diary survey, with 2 possible high-compliance bonuses of US $5, one if participants completed at least 6 daily surveys during the first week and one if participants completed at least 12 daily surveys during the second and third weeks combined. Participants could earn up to US $67 with high study compliance. Additionally, participants received 1 raffle ticket for each high-compliance bonus, for a 1 in 100 chance of winning a US $50 gift card for high compliance in the first week and a 1 in 100 chance of winning a US $100 gift card for high compliance in the second and third weeks. Of the 2012 students who participated in the daily diary study, 1599 (79.5%) completed at least 6 of the first 7 days, and 1284 (63.8%) completed at least 12 of the last 14 days.

### Surveys

#### Baseline Survey

Participants who completed the 15-minute baseline survey were automatically enrolled in a 21-day diary study. The baseline survey assessed a variety of topics, including demographics, college standing, health, COVID-19 prevention behaviors and beliefs, housing and food insecurity, antiracism behavior, experiences of bias or discrimination, and substance use.

#### Demographic, Individual, and Family Characteristics

In the first part of the survey, general demographic questions were asked, including questions about participants’ age, sex, gender, sexual or gender minority status, race or ethnicity, height, weight, current health conditions, and employment status during the academic year. Participants reported on their high school grade point average, recent and cumulative college grade point average, country of origin, political ideology, whether their permanent home was in or out of state, and their parents’ highest degree (used to identify first-generation college students). Participants also reported on several different aspects of their relationship with their caregivers, including family cohesion and attachment, family conflict, identification with parents, and conflict behavior [8-11].

#### College-Specific Characteristics

Participants reported their class standing and were asked about their group affiliations in college (honors program, Greek system, other philanthropic organizations), previous participation in the university’s early start program, and current learning modes. During the spring survey, participants could participate in remote classroom lectures. The majority of classes were reinstated in person for the duration of the fall survey; however, a remote option was often available as well. Participants were also asked about their use of on-campus health services and their feelings of belonging at the university.

#### Food and Housing Insecurity

Food insecurity was assessed by a validated 2-item screening instrument to assess participants’ access to enough food every day. The screener asked about worries around food running out and not having enough money to buy more food [12]. Students’ housing security, homelessness, and living situation were assessed with modules from the Researching Basic Needs in Higher Education instruments [13].

#### General Experiences of Discrimination and Antiracist Behavior

Participants completed the 21-item Anti-racism Behavioral Inventory [14], which included items such as “When I hear people telling racist jokes and using negative racial stereotypes, I usually confront them.” and “The police unfairly target Black men and Latinos.” Participants also completed the 7-item Williams Everyday Discrimination Scale [15], which assessed perceptions such as people acting as if they think the individual is not smart or as if they are afraid of the individual.

#### Mental Health

Depression symptoms were assessed using the 10-item Center for Epidemiologic Studies Depression Scale (CESD-R-10) [16]. Anxiety symptoms were assessed using the 6-item General Anxiety subscale of the Center for Collegiate Mental Health Instrument (CCAPS-34) [17]. Social anxiety was assessed using the 6-item Social Interaction Anxiety Scale (SIAS-6) [18]. The adult attention-deficit/hyperactivity disorder (ADHD) Self-Report Scale (ASRS, version 1.1) [19] was used to assess adult symptoms of ADHD. Participants also completed the 16-item Social Responsiveness Scale (SRS-2-S) [20] and an 8-item instrument to assess their level of flourishing [21].

#### Behavior

Students’ histories of alcohol, cannabis, nicotine (including vaping, cigarettes, and other tobacco products), and other illicit substance use behaviors were assessed, as was their perception of alcohol use on campus [22]. Recent sexual activity was assessed.

#### 21-Day Daily Diary Survey

Each morning, beginning the day after completing the baseline survey, participants were sent a link to that day’s 5-minute survey. Participants were required to click on the link to initiate the daily survey by midnight and had until 1:00 AM to complete the survey. Daily diary surveys asked in-depth questions about substances used the day before (alcohol, marijuana, smoking, and other illicit substances) and their consequences, health behaviors, sleep, pleasurable experiences, and feelings of belonging, engagement, and well-being, as well as COVID-19 prevention behaviors. If participants did not report any substance use the day before, they were asked an alternative set of questions about food and housing insecurity and physical activity instead of the in-depth questions about substance use and consequences.

#### Substance Use, Consequences, and Context of Use

Participants were asked if they used a substance (ie, alcohol, marijuana, cigarettes, prescription painkillers, heroin, psychedelics, sedatives, or stimulants). If they answered yes to any of the substances, they were asked follow-up questions regarding how much of each they took. If they answered yes to alcohol or marijuana, they were asked a follow-up question about impairment [23,24]. If a participant reported any substance use, they were asked about any positive (eg, feeling buzzed, more energetic) or negative consequences (eg, blacking out or feeling nauseated) of using those substances [25]. For each substance participants reported using, they were asked where they used the substance (eg, home, a friend’s house, and outdoors). They also reported if they used that substance around other people, and answered follow-up questions about who those people were.

#### Daily Experiences of Discrimination

Discrimination was asked with 1 question, adapted from the Williams Daily Discrimination Scale, assessing how strongly they experienced bias or discrimination yesterday on a scale from 0=not at all to 5=very strongly [15].

#### Mental Health

Daily stress was assessed on a 0-100 scale from 0=not at all to 100=very extremely, with follow-up questions asking if anything COVID-19–related increased that stress. Participants were then asked to rate how stressful school relationships with friends or roommates and family interactions were. Daily affect was assessed using a 10-item scale assessing different feelings and emotions. This experience sampling method affect scale, taken from Wichers et al [26], was modeled after the Positive and Negative Affect Schedule Instrument [27], specifically selecting high-loading items on the negative affect (ie, insecure, lonely, anxious, low, guilty, and suspicious) and positive affect (ie, cheerful, content, energetic, and enthusiastic) spectra. Participants also answered 2 questions assessing worry (ie, I worried what other people thought of me and I was worried that I would say or do the wrong things), drawn from the social anxiety scale from Kashdan and Steger [28].

#### Pleasure, Belonging, and Well-Being

Experiences of pleasure were measured with 3 questions asking participants if they had any pleasure in activities yesterday and, if so, what type of activities they were and with what intensity they experienced pleasure [29]. Belonging questions asked the extent to which the participant felt like they belonged in the university environment [30]. Participants were asked 5 questions from the flourishing scale about whether they led a purposeful or meaningful life and were satisfied with their life, bored, or engaged [21].

#### Eating and Food Insecurity

Participants reported where they ate yesterday. To assess food insecurity, they were asked whether they were worried about paying for food tomorrow and what their food situation was the day before (food insecurity screening instrument) [12]. To help balance the questionnaire length across all days, participants were asked these questions only on days when they reported no substance use.

#### Sleep and Physical Activity

Each day, participants reported the time they went to bed, the time they woke up, and where they slept the night before (eg, college housing, apartment, and family home). Physical activity was assessed by 3 questions asking about vigorous and moderate-intensity exercise and sitting and reclining time. Participants were asked these questions only on days when they reported no substance use.

### Data Analysis

#### Scale Construction for Baseline Constructs

For the *depression scale* (based on the CESD-10), 10 items with choices ranging from 0=rarely to 3=most of the time were summed to create a depression score, which ranged from 0 to 30. For the *anxiety scale* (based on CCAPS-34), 6 items with choices ranging from 0=not at all like me to 4=extremely like me were averaged to create an anxiety score, which ranged from 0 to 4. For the *social anxiety scale* (based on SIAS-6B), 6 items with choices ranging from 0 (not at all) to 4 (extremely) were summed to create a social anxiety score, which ranged from 0 to 24. The *ADHD symptoms scale* (based on ASRS_part A) was based on 6 items with response options from 0 (never) to 4 (very often); these items were converted to binary variables, wherein 3 of the items were scored as 1 if their value was greater than 1 (0 otherwise) and the remaining 3 items were scored as 1 if their value was greater than 2 (0 otherwise) [19,31]. The sum of these binary indicators created the ADHD symptoms scale, with a possible range of 0-6. For the *social responsiveness score* (based on the SRS-2-S), 16 items with response options from 0 (not true) to 3 (almost always true) were summed to create an SRS-2-S score, which ranged from 0 to 48. The original SRS-2 has 65 items, and scores could range from 0 to 195. The SRS-2-S scores were converted to an estimated SRS-2 score based on the recommended conversion table [32]. This estimated score could range from 5.7 (corresponding to SRS-2-S values of 0) to 181.5 (corresponding to SRS-2-S values of 48). For the *flourishing scale* at baseline, 8 items with response options ranging from 1=strongly disagree to 7=strongly agree were summed. The 8 items on this scale had high reliability (Cronbach α=0.98). To calculate the *closeness with caregivers scale*, 4 items with response options ranging from 0 (almost never) to 100 (almost always) were averaged. The items asked about whether caregivers respected participants’ feelings and encouraged them to talk about difficulties and whether they feel close to caregivers and tell caregivers about problems. This scale had high reliability (Cronbach α=0.87).

#### Scale Construction for Daily Constructs

For each observed day, the following scale scores were created. In total, 3 items tailored to assess aspects of flourishing in daily life were averaged to create a *daily flourishing scale*: All things considered, I am satisfied with my life yesterday; I was engaged and interested in my activities yesterday; and I led a purposeful and meaningful life yesterday. Each item had response options of 1=strongly disagree to 7=strongly agree. A *daily positive affect score* and a *daily negative affect score* were calculated using items from a 10-item scale where choices ranged from 1=never to 7=always [26]. In total, 4 of the items were averaged to create a positive affect score, and the remaining 6 items were averaged to create a negative affect score. A *daily worry score* was calculated by averaging 2 items that asked participants the extent to which they worried about what others thought of them and the extent to which they worried they would say or do the wrong things. Response options ranged from 1=not at all or slightly to 5=extremely. This scale had high reliability (Cronbach α=0.88).

#### Statistical Modeling

Descriptive statistics were calculated for a variety of constructs at baseline and in the daily study. Then, variance in daily response variables due to between- versus within-person sources was partitioned using multilevel models, with a separate intercept-only linear mixed model specified for each variable. In the case of a discrete outcome, this estimated percentage is an approximation; this is a reasonable approximation when the underlying probability of the response is not close to 0 or 1 [33]. All descriptive statistics and multilevel models were conducted using R (version 4.1.2; The R Foundation).

## Results

A summary of descriptive statistics from the baseline assessment appears in Table 1, and Table 2 presents descriptive statistics from the daily surveys, along with the proportion of variance that can be attributed to between-person versus person-level sources of variability.

**Table 1:** Descriptive statistics from the baseline survey.

Baseline measure			Study sample (N=2068)		Population of UP^a^ students (N=39,392)
Identify as female gender, n (%)			1378 (66.6)		18,639 (47.3)
**Race or ethnicity, n (%)**					
	Hispanic ethnicity	162 (7.8)		3029 (7.7)	
	Non-Hispanic Black	56 (2.7)		1713 (4.3)	
	Non-Hispanic White	1433 (69.3)		25,674 (65.2)	
	Non-Hispanic Asian	307 (14.8)		2651 (6.7)	
	Other and multiple race categories	110 (5.3)		6325 (16.1)	
**Class standing, n (%)**					
	Lower undergraduates (first or second year)	1039 (50.2)		18,192 (46.2)	
	Upper undergraduates (third year or later)	1025 (49.6)		21,200 (53.8)	
	Foreign-born student, n (%)	284 (13.7)		3988 (10.1)^b^	
	First-generation student, n (%)	348 (16.8)		6621 (16.8)	
	In-state student status, n (%)	1330 (64.3)		23,289 (59.1)	
	Age (years), mean (SD)	19.8 (1.3)		—^b^	
**Employment status, n (%)**					
	Full-time	20 (1.0)		—	
	Part-time	771 (37.3)		—	
	Not employed	1234 (59.7)		—	
	Sexual or gender minority, n (%)	361 (17.5)		—	
	Engaged with Greek system, n (%)	286 (13.8)		—	
	Food insecure (past month), n (%)	395 (19.1)		—	
	Housing insecure (past month), n (%)	170 (8.2)		—	
	Past-month alcohol use, n (%)	1316 (63.6)		—	
	Past-month heavy episodic drinking, n (%)	419 (20.3)		—	
	Past-month vaping, n (%)	372 (18.0)		—	
	Past-month cigarette use, n (%)	127 (6.1)		—	
	Past-month cannabis use, n (%)	378 (18.3)		—	
	High school GPA^c^, median (IQR)	3.9 (0.4)		—	
	Cumulative college GPA, mean (SD)	3.5 (0.4)		—	
	Antiracism behavior: scale of 21-105, mean (SD)	67.7 (15.6)		—	
	General experiences of discrimination: scale of 1=almost daily to 6=never, mean (SD)	4.85 (0.9)		—	
	Depression: scale of 0-30, mean (SD)	11.0 (6.4)		—	
	General anxiety: scale of 0-4, mean (SD)	1.40 (1.0)		—	
	Social anxiety: scale of 0-24, mean (SD)	8.5 (6.0)		—	
	ADHD^d^ symptoms: scale of 0-6, mean (SD)	2.5 (1.8)		—	
	Social responsiveness: scale of 5.7-181.5, mean (SD)	49.4 (25.6)		—	
	Flourishing: scale of 8-56, mean (SD)	45.3 (7.4)		—	
	Closeness with caregivers: scale of 0-100, mean (SD)	74.0 (20.7)		—	

^a^UP: University Park.

^b^Not applicable.

^c^GPA: grade point average.

^d^ADHD: attention-deficit/hyperactivity disorder.

**Table 2:** Descriptive statistics from daily diary study (based on 33,722 total person-days from n=2012 participants).

Daily measure	Values	Proportion of variance, %	
		Between-person	Within-person
Alcohol use day	4902 (14.5)^a^	19.4%	80.6%
Heavy episodic drinking day	2453 (7.3)^a^	15.5%	84.5%
Vaping day	1888 (5.6)^a^	69.8%	30.2%
Cigarette use day	254 (0.8)^a^	40%	60%
Cannabis use day	1846 (5.5)^a^	56%	44%
Other illicit substance use day	879 (2.6)^a^	70.1%	29.9%
Experienced interest or pleasure in activity	24,771 (73.5)^a^	40.7%	59.3%
Stress: how stressful was yesterday on scale of 0=not at all to 100=extremely	36.3 (27.2)^b^	37.4%	62.6%
How strongly experienced bias or discrimination: 0=not at all to 5=very strongly	1.17 (0.5)^b^	52.3%	47.7%
Positive affect: 1=never to 7=always	3.68 (1.5)^b^	57.5%	42.5%
Negative affect: 1=never to 7=always	2.28 (1.1)^b^	63.6%	36.4%
Worried: 1=not at all or slightly to 5=extremely	2.16 (1.1)^b^	67.7%	32.3%
Feelings of belonging at the university: 1=strongly disagree to 7=strongly agree	5.1 (1.6)^b^	70.7%	29.3%
Flourishing: 1=strongly disagree to 7=strongly agree	4.63 (1.5)^b^	60.2%	29.8%
Poor food situation in past 24 hours^c^	6103 (23.5)^b^	55.9%	44.1%
Worried about how to pay for food tomorrow^c^	855 (3.3)^b^	52%	48%
Slept in own dorm or room or apartment or family home last night^c^	24,554 (94.4)^b^	29.8%	70.2%
Engaged in moderate or vigorous physical activity^c^	22,095 (84.9)^b^	44.8%	55.2%

^a^Values are n (%). Estimated percentages of variance are based on intraclass correlation coefficients in multilevel models; these numbers are approximations in the case of binary response variables.

^b^Values are mean (SD).

^c^Questions only asked on non–substance use days; thus, percentages are based on 26,020 days.

### Participant Characteristics at Baseline

A total of 2068 students completed the baseline survey, including 1378 (66.6%) female students. The participants’ ages ranged from 18 to 24 (mean 19.8, SD 1.3) years at baseline. The sample included 1039 lower-level undergraduates (50.2% in the first or second year) and 1025 upper-level undergraduates (49.6% in the third year or later).

#### Sample Versus Population Characteristics

Table 1 shows descriptive statistics for the data about the population of 39,392 undergraduate students. Data are from the Penn State data digest [34], compiled for the fall 2021 semester. The racial-ethnic composition of the sample (7.8% Hispanic or Latinx, 2.7% non-Hispanic Black, 69.3% non-Hispanic White, 14.8% non-Hispanic Asian, and 5.3% other or mixed races) was similar to that of the population of students at this campus (7.7% Hispanic or Latinx, 4.3% non-Hispanic Black, 65.2% non-Hispanic White, 6.7% non-Hispanic Asian, and 16.1% other or mixed races). The sample had a larger proportion of female students than the broader university population (47.3% in population), and slightly higher percentages of lower-level students (50.2% vs 46.2% in the population), foreign-born students (13.7% vs 10.1%), and in-state students (64.3% vs 59.1%). The proportion of first-generation students was 16.8% in both the sample and the population.

#### Additional Sample Characteristics

Of participants who completed the baseline survey, 38.3% (n=791) were employed, 17.5% (n=361) identified as a sexual or gender minority, 13.8% (n=286) were affiliated with the Greek system, 19.1% (n=395) reported food insecurity, and 8.2% (n=170) reported housing insecurity. The prevalence of past-month substance use behaviors was 63.6% (n=1316) for any alcohol use, 20.3% (n=419) for heavy episodic drinking (ie, binge drinking), 18% (n=372) for vaping, 6.1% (n=127) for cigarette use, and 18.3% (n=378) for cannabis use. The median high school grade point average was 3.9 with an IQR of 0.4, and their mean cumulative grade point average in college was 3.5 (SD 0.4). Mean scale scores for antiracism behavior, discrimination, depression, general anxiety, social anxiety, ADHD symptoms, social responsiveness, flourishing, and closeness with caregivers are provided in Table 1.

### Student Health and Well-Being in Daily Life

On 21 consecutive days, participants were prompted to complete a survey that assessed a comprehensive set of daily markers of health, health behaviors, and well-being. Students completed the daily survey in an average of 16.8 (SD 5.7) days.

#### Substance Use, Stress, Discrimination, Affect, and Flourishing

Of the 33,722 person-days, a minority were substance use days; 4902 days (14.5%) involved alcohol use, 2453 days with heavy episodic drinking (7.3%), 1888 days with vaping (5.6%), 254 days with cigarettes (0.8%), 1846 days with cannabis (5.5%), and 879 days with other illicit substance use (2.6%). Experiences of pleasure were reported on 24,771 of 33,722 days (73.5%). Mean scale scores for daily stress, experiences of bias or discrimination, positive affect, negative affect, worry, feelings of belonging, and flourishing are provided in Table 2.

#### Additional Indicators of Well-Being

Based on data from the 26,020 person-days with no reported substance use, a poor food situation was reported on 6103 days (23.5%); worry about how to pay for food tomorrow was reported on 855 (3.3%) days; students slept in their own dormitory, room, apartment, or family home on 24,554 (94.4%) days; and students engaged in moderate or vigorous physical activity on 22,095 (84.9%) days.

#### Between- Versus Within-Person Variability

Partitioning variance into between- versus within-person sources of variance for daily indicators of health and well-being provides insight into which constructs tend to be more person-specific and potentially explainable by person-level characteristics, as opposed to those that are more day-specific and potentially explainable by the contextual characteristics of a particular day. Between-person differences explained the majority of variance in daily vaping (69.8% attributable to between-person differences), cannabis use (56%), other illicit substance use (70.1%), experiences of bias or discrimination (52.3%), positive affect (57.5%), negative affect (63.6%), worry (67.7%), feelings of belonging at the university (70.7%), flourishing (60.2%), poor food situation (55.9%), and worry about how to pay for food (52%).

In contrast, within-person differences (ie, day-to-day fluctuations within individuals) explained the majority of the variance in daily alcohol use (80.6% attributable to within-person differences), heavy episodic drinking (84.5%), cigarette use (60%), experiences of pleasure (59.3%), stress (62.6%), where a student slept last night (70.2%), and physical activity (55.2%), indicating that day-level contextual factors may be more relevant for these daily experiences and behaviors.

## Discussion

### Principal Results

This study examined a wide range of health and well-being indicators among college students in daily life during the COVID-19 pandemic. Consistent with prior research on substance use in daily life, the proportion of variance attributed to within-person fluctuations was particularly high for daily alcohol use, heavy episodic drinking, and cigarette use, suggesting that these behaviors are highly sensitive to contextual characteristics [35-38]. In contrast, daily vaping behavior and other illicit substance use behavior tended to be most stable as person-level characteristics; this is consistent with recent research that has shown level of dependence to be positively associated with stability of behavior across days [39]. Variance in other aspects of daily mental health (belonging, flourishing, positive affect, negative affect, and worry) was also primarily attributable to between-person (ie, trait-like) differences. This finding is consistent with a coordinated analysis of 6 intensive longitudinal studies of positive and negative affect; variance decomposition of daily data reliably showed affect to be more strongly characterized by between-person differences [40].

These findings suggest where student programming and intervention efforts may be most effectively appropriated in this population. For example, available mental health services and interventions directed to certain individuals who would benefit most may be the best strategy for resource allocation on college campuses, whereas the provision of just-in-time mental health interventions on days of particularly high negative affect or worry or low positive affect may be less effective. In contrast, interventions designed to reduce problematic alcohol use and cigarette use may be best targeted toward risky contexts that students move in and out of throughout their days.

This type of intensive surveillance provides data that characterize not just population-average levels on aspects of health and well-being and their correlates, but also sheds light on day-to-day variability in student health and well-being and dynamic contexts that may place students at heightened risk for poor outcomes on a given day. By partitioning the variance of daily indicators of health and well-being, this study design provides the opportunity to determine which indicators of health and well-being are more trait-like and which are more state-like.

### Limitations and Future Work

One limitation of this study was the low rate of participation. This may produce nonresponse bias, which has been documented among college students, with survey respondents more likely to be female, socially engaged, and financially secure, with differences in personality compared to nonresponders (eg, [41]). Another limitation of this study was that rolling recruitment spanned from February through November 2020 (with no recruitment during the summer term); thus, students participated in the study at qualitatively distinct times during the COVID-19 pandemic. Most notably, vaccinations became widely available in mid-2020, which could have impacted student mental health and social behaviors. Thus, it may be important to determine whether these findings replicate in future analyses using daily diary data collected after the pandemic.

### Conclusions

Certain daily experiences and behaviors related to college students’ health and well-being, such as vaping, negative affect, and feelings of belonging at one’s university, distinguish at-risk individuals from other students. In contrast, other experiences and behaviors, such as alcohol use and cigarette use, stress, and physical activity, may be driven more by contextual risk factors operating on certain days. These findings suggest that some aspects of health and well-being may be most amenable to services that target students more generally, while others may be more amenable to interventions that target particularly risky students, days, or contexts.

Daily diary studies can inform practical policy recommendations to higher-education institutions, enabling them to make data-informed decisions on university closures and the development of programs to support student health and well-being. This approach is essentially a community-based surveillance system that can provide insight into student characteristics and contextual factors that place individuals at risk for negative outcomes.

## Abbreviations

ADHD: attention-deficit/hyperactivity disorder
ASRS: Adult ADHD Self-Report Scale
CCAPS-34: Center for Collegiate Mental Health Instrument
CESD-R-10: Center for Epidemiologic Studies Depression Scale
REDCap: Research Electronic Data Capture
SIAS-6: Social Interaction Anxiety Scale
SRS-2-S: Social Responsiveness Scale

## Data Availability

The data sets generated and analyzed during this study are available from the corresponding author on reasonable request.

## References

[1] (2020). Center for Collegiate Mental Health 2019 Annual Report: Global Health Education and Learning Incubator at Harvard University.

[2] NA (2020). Key substance use and mental health indicators in the United States: results from the 2019 national survey on drug use and health. Substance Abuse and Mental Health Services Administration.

[3] White A, Hingson R (2013). The burden of alcohol use: excessive alcohol consumption and related consequences among college students. Alcohol Res Curr Rev.

[4] Czeisler MÉ, Lane RI, Petrosky E, Wiley JF, Christensen A, Njai R, Weaver MD, Robbins R, Facer-Childs ER, Barger LK, Czeisler CA, Howard ME, Rajaratnam SMW (2020). Mental health, substance use, and suicidal ideation during the COVID-19 pandemic - United States, June 24-30, 2020. MMWR Morb Mortal Wkly Rep.

[5] Wang X, Hegde S, Son C, Keller B, Smith A, Sasangohar F (2020). Investigating mental health of US college students during the COVID-19 pandemic: cross-sectional survey study. J Med Internet Res.

[6] Gopalan M, Linden-Carmichael A, Lanza S (2022). College students' sense of belonging and mental health amidst the COVID-19 pandemic. J Adolesc Health.

[7] Lanza ST, Whetzel CA, Linden-Carmichael AN, Newschaffer CJ (2022). Change in college student health and well-being profiles as a function of the COVID-19 pandemic. PLoS One.

[8] Bloom BL (1985). A factor analysis of self-report measures of family functioning. Fam Process.

[9] Furstenberg FF, Cook TD, Eccles J, Elder GH (2000). Managing to Make It: Urban Families and Adolescent Success.

[10] Robin AL, Foster SL (2002). Negotiating Parent-Adolescent Conflict: A Behavioral-Family Systems Approach.

[11] Armsden GC, Greenberg MT (1987). The inventory of parent and peer attachment: individual differences and their relationship to psychological well-being in adolescence. J Youth Adolesc.

[12] Hager ER, Quigg AM, Black MM, Coleman SM, Heeren T, Rose-Jacobs R, Cook JT, Ettinger de Cuba SA, Casey PH, Chilton M, Cutts DB, Meyers AF, Frank DA (2010). Development and validity of a 2-item screen to identify families at risk for food insecurity. Pediatrics.

[13] Crutchfield R, Maguire J (2017). Researching Basic Needs in Higher Education: Qualitative and Quantitative Instruments to Explore a Holistic Understanding of Food and Housing Insecurity.

[14] Pieterse AL, Utsey SO, Miller MJ (2015). Development and initial validation of the anti-racism behavioral inventory (ARBI). Couns Psychol Q.

[15] Williams DR, Yu Y, Jackson JS, Anderson NB (1997). Racial differences in physical and mental health: socio-economic status, stress and discrimination. J Health Psychol.

[16] Zhang W, O'Brien N, Forrest JI, Salters KA, Patterson TL, Montaner JSG, Hogg RS, Lima VD (2012). Validating a shortened depression scale (10 item CES-D) among HIV-positive people in British Columbia, Canada. PLoS One.

[17] Locke BD, McAleavey AA, Zhao Y, Lei PW, Hayes JA, Castonguay LG, Li H, Tate R, Lin Y (2017). Development and initial validation of the counseling center assessment of psychological symptoms–34. Meas Eval Couns Dev.

[18] Peters L, Sunderland M, Andrews G, Rapee RM, Mattick RP (2012). Development of a short form social interaction anxiety (SIAS) and social phobia scale (SPS) using nonparametric item response theory: the SIAS-6 and the SPS-6. Psychol Assess.

[19] Kessler RC, Adler L, Ames M, Demler O, Faraone S, Hiripi E, Howes MJ, Jin R, Secnik K, Spencer T, Ustun TB, Walters EE (2005). The world health organization adult ADHD self-report scale (ASRS): a short screening scale for use in the general population. Psychol Med.

[20] Constantino JN, Gruber CP (2012). Social Responsiveness Scale: SRS-2.

[21] Diener E, Wirtz D, Tov W, Kim-Prieto C, Choi D, Oishi S, Biswas-Diener R (2010). New well-being measures: short scales to assess flourishing and positive and negative feelings. Soc Indic Res.

[22] Graupensperger S, Turrisi R, Jones D, Evans MB (2020). Longitudinal associations between perceptions of peer group drinking norms and students' alcohol use frequency within college sport teams. Alcohol Clin Exp Res.

[23] Cloutier RM, Calhoun BH, Lanza ST, Linden-Carmichael AN (2022). Assessing subjective cannabis effects in daily life with contemporary young adult language. Drug Alcohol Depend.

[24] Linden-Carmichael AN, Calhoun BH (2022). Measuring subjective alcohol effects in daily life using contemporary young adult language. Exp Clin Psychopharmacol.

[25] Lee CM, Cronce JM, Baldwin SA, Fairlie AM, Atkins DC, Patrick ME, Zimmerman L, Larimer ME, Leigh BC (2017). Psychometric analysis and validity of the daily alcohol-related consequences and evaluations measure for young adults. Psychol Assess.

[26] Wichers M, Peeters F, Rutten BPF, Jacobs N, Derom C, Thiery E, Delespaul P, van Os J (2012). A time-lagged momentary assessment study on daily life physical activity and affect. Health Psychol.

[27] Watson D, Clark LA, Tellegen A (1988). Development and validation of brief measures of positive and negative affect: the PANAS scales. J Pers Soc Psychol.

[28] Kashdan TB, Steger MF (2006). Expanding the topography of social anxiety. An experience-sampling assessment of positive emotions, positive events, and emotion suppression. Psychol Sci.

[29] Heininga VE, van Roekel E, Ahles JJ, Oldehinkel AJ, Mezulis AH (2017). Positive affective functioning in anhedonic individuals' daily life: anything but flat and blunted. J Affect Disord.

[30] Walton GM, Cohen GL (2007). A question of belonging: race, social fit, and achievement. J Pers Soc Psychol.

[31] Adler L, Kessler R, Spencer T (2003). Adult ADHD Self-Report Scale-v1. 1 (ASRS-v1. 1) Symptom Checklist.

[32] Lyall K, Hosseini M, Ladd-Acosta C, Ning X, Catellier D, Constantino JN, Croen LA, Kaat AJ, Botteron K, Bush NR, Dager SR, Duarte CS, Fallin MD, Hazlett H, Hertz-Picciotto I, Joseph RM, Karagas MR, Korrick S, Landa R, Messinger D, Oken E, Ozonoff S, Piven J, Pandey J, Sathyanarayana S, Schultz RT, St John T, Schmidt R, Volk H, Newschaffer CJ, program collaborators for Environmental influences on Child Health Outcomes (2021). Distributional properties and criterion validity of a shortened version of the social responsiveness scale: results from the ECHO program and implications for social communication research. J Autism Dev Disord.

[33] Goldstein H, Browne W, Rasbash J (2002). Partitioning variation in multilevel models. Understanding statistics: statistical issues in psychology, education, and the social sciences. Sciences.

[34] (2021). Penn State Data Digest.

[35] Maggs JL, Williams LR, Lee CM (2011). Ups and downs of alcohol use among first-year college students: number of drinks, heavy drinking, and stumble and pass out drinking days. Addict Behav.

[36] Colder CR, Lloyd-Richardson EE, Flaherty BP, Hedeker D, Segawa E, Flay BR, Tobacco Etiology Research Network (2006). The natural history of college smoking: trajectories of daily smoking during the freshman year. Addict Behav.

[37] Park CL, Armeli S, Tennen H (2004). The daily stress and coping process and alcohol use among college students. J Stud Alcohol.

[38] Piasecki TM, Jahng S, Wood PK, Robertson BM, Epler AJ, Cronk NJ, Rohrbaugh JW, Heath AC, Shiffman S, Sher KJ (2011). The subjective effects of alcohol-tobacco co-use: an ecological momentary assessment investigation. J Abnorm Psychol.

[39] Buu A, Cai Z, Li R, Wong SW, Lin HC, Su WC, Jorenby DE, Piper ME (2021). Validating e-cigarette dependence scales based on dynamic patterns of vaping behaviors. Nicotine Tob Res.

[40] Scott SB, Sliwinski MJ, Zawadzki M, Stawski RS, Kim J, Marcusson-Clavertz D, Lanza ST, Conroy DE, Buxton O, Almeida DM, Smyth JM (2020). A coordinated analysis of variance in affect in daily life. Assessment.

[41] Porter SR, Whitcomb ME (2005). Non-response in student surveys: the role of demographics, engagement and personality. Res High Educ.

